# Bovine adipose mitochondrial adaptation and a potential lactate–ketone toggle in early lactation

**DOI:** 10.3389/fvets.2025.1676955

**Published:** 2025-12-03

**Authors:** Nial J. O’Boyle, Miguel Chirivi, Ursula Abou-Rjeileh, David Salcedo-Tacuma, José M. dos Santos Neto, Crystal Prom, Jair Parales-Girón, Adam L. Lock, Reinhard Stöger, Lisa Chakrabarti, G. Andres Contreras

**Affiliations:** 1 School of Veterinary Medicine and Science, University of Nottingham, Nottingham, United Kingdom; 2 Department of Large Animal Clinical Sciences, Michigan State University, East Lansing, MI, United States; 3 Department of Biochemistry and Molecular Medicine, West Virginia University School of Medicine, Medical Center Drive, Morgantown, WV, United States; 4 Department of Animal Science, Michigan State University, East Lansing, MI, United States; 5 School of Biosciences, University of Nottingham, Nottingham, United Kingdom

**Keywords:** mitochondria, bovine, adipose, transcriptomics, lactate, ketone, peripartum

## Abstract

The periparturient period creates an intense energy demand due to the onset of lactation, which requires substantial glucose for milk synthesis, particularly in high producing cows, contributing to a high incidence of postpartum metabolic disease. We explored the transcriptomic adaptation of subcutaneous adipose tissue (AT), with a specific focus on metabolic gene networks and the mitochondrial component. Mitochondria coordinate cellular energy dynamics by linking the oxidation of nutrients to ATP synthesis via oxidative phosphorylation (OXPHOS). However, their role in postpartum metabolic disease is not clear. We therefore re-analysed a longitudinal RNA-seq dataset of subcutaneous AT from 12 healthy multiparous Holstein cows, sampled pre-calving and at two early-lactation time-points, to explore mitochondrial pathways. This analysis revealed downregulation of differentially expressed genes (DEGs), encoding components of the electron transport system and OXPHOS, in the postpartum phase, concurrent with a shift to DEGs associated with glycolysis. Given the observed glycolytic shift, an analysis of plasma lactate during the periparturient period was undertaken, to explore how this glycolysis-derived substrate fluctuates in this altered metabolic state. A postpartum decline in plasma lactate, alongside rising *β*-hydroxybutyrate, was further demonstrated in clinical ketotic cows, revealing a potential metabolic toggle between lactate and ketones; aligning with the concept that fuel sources will alter depending on redox and metabolic conditions. This supports the emerging view that ketones are not merely pathological markers but may serve as adaptive metabolic signals, warranting further investigation into their role in dairy cow metabolism. Further understanding of how mitochondria function during this energy-intensive postpartum phase of the dairy cow, may help elucidate how adipose tissue contributes to metabolic resilience or perturbation during early lactation.

## Background

The periparturient period represents a challenging time for the dairy cow, with high incidences of metabolic-related disease affecting nearly half of all high-yielding cows (1). Only 15.4% of the 270 million global dairy cows provide 45.4% of milk output (2). This skewed production demonstrates the added metabolic pressure on high-yielding cows. Compounding the normal mammalian transitions from gestation to the onset of lactation, is a high energy requirement for milk production. Feed intake often fails to meet the energy requirements in the weeks postpartum, resulting in a negative energy balance (3). To meet energy demands, adipose tissues (AT) mobilise fatty acid (FA) reserves via lipolysis (4). When lipolysis is excessive, it can give rise to an inflammatory state with concurrent oxidative stress, and excess remodelling of adipose tissue (4). This state is also correlated with a rise in blood ketones (hyperketonaemia), which can progress to ketosis and clinical symptoms, including reduced milk production, impaired reproductive performance, and increased risk of infectious disease (1).

It is possible to gain an understanding of metabolic changes in an animal by examining cellular processes that are involved in the production of energy. Mitochondria are cellular organelles found in almost all eukaryotic cells. Mitochondria regulate cellular respiration, maintain redox balance, buffer intracellular calcium, generate metabolic intermediates, initiate apoptotic signalling (5), and also produce chemical energy in the conversion of ADP to ATP, utilising oxygen for the process (5). A variety of substrates can be used for ATP production including FA (5). In the dairy cow with high lipolysis rates mitochondrial oxidative capacity can be overwhelmed, leading to the accumulation of intermediates and excess reactive oxidant species (ROS) (6). This further amplifies inflammation through cytokine release and macrophage recruitment (7). While these pathways are well described in rodent and human models, similar mitochondrial-inflammatory mechanisms have also been demonstrated in bovine adipose tissue (8). However, how bovine adipose mitochondria adapt, or fail to adapt, during the periparturient period remains poorly understood.

Lactate (produced in the cytosol but oxidised in mitochondria) and ketone bodies (mostly synthesised in hepatic mitochondria and oxidised in the mitochondrial matrix of peripheral tissues) are two key metabolites in the context of adaptation, with lactate recognised as a signalling molecule linking glycolysis and OXPHOS (9). Ketone metabolism, in contrast, depends entirely on mitochondrial oxidation and sufficient oxygen availability (10). Both play roles in energy flexibility: lactate dominates when glycolytic flux is high, while ketones take precedence during prolonged energy stress (9, 10). The mobilisation and use of ketones are well described in early lactation, providing an energy source during periods of low glucose availability, particularly under intensive lipolysis (1). However, the role of lactate in bridging energy shortfalls in the bovine, and how these pathways intersect with mitochondrial control, remains less well defined.

Building on a previous analysis of dairy cow AT transcriptome (11), which provided a detailed exploration of lipolysis, inflammation, and tissue remodelling, our study now investigates mitochondrial adaptation. While FA oxidation is known to be a limiting factor in successful dairy cow transition, the reasons for the inadequate oxidative capacity remain unclear (6, 12). By focusing on transcriptomic changes that may influence mitochondrial function, we aimed to explore whether adaptations related to oxidative capacity, immune-associated gene expression, and energy substrate flexibility were evident.

## Methods

### Samples

Data from a longitudinal cohort study on 12 healthy multiparous Holstein cows at Michigan State University Dairy Cattle Teaching and Research Center were re-analysed. The original data are available in the NCBI Gene Expression Omnibus (accession number: GSE159224) (11).

Weekly body condition scores (BCS) were assessed (13), and cows were categorised by BCS, previous lactation yield, and parity. Subcutaneous adipose tissue (SCAT) samples were collected at three time points as reported by Abou-Rjeileh et al. (14): 11 ± 3.6 days prepartum (PreP), and 6 ± 1 day postpartum (PP1) and 13 ± 1.4 days postpartum (PP2). RNA was extracted from SCAT for transcriptomic analysis, as described by Salcedo-Tacuma et al. (11).

### Initial data preparation

**Figure 1:**
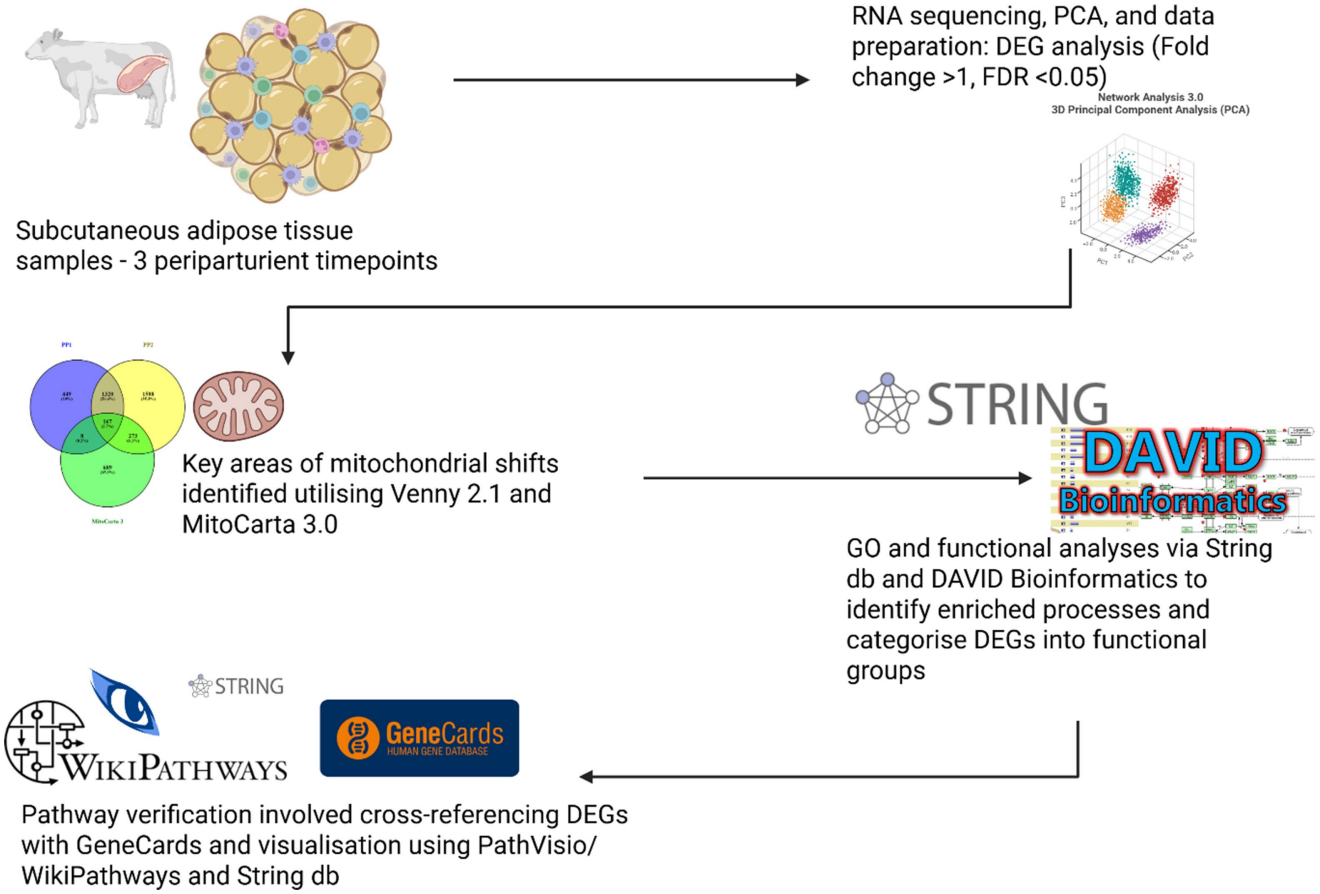
Workflow for transcriptomic analysis of periparturient adipose tissue. Adipose tissue samples were collected at three timepoints. RNA sequencing and PCA identified differentially expressed genes (DEGs) based on fold change (>1) and statistical significance (FDR < 0.05). Key mitochondrial changes were mapped using MitoCarta 3.0 and Venny 2.1. GO Terms analyses and Functional enrichment were performed using STRING-db and DAVID to classify DEGs into biological pathways. Pathway validation involved cross-referencing with GeneCards and visualising networks through PathVisio and WikiPathways.

**Figure 2:**
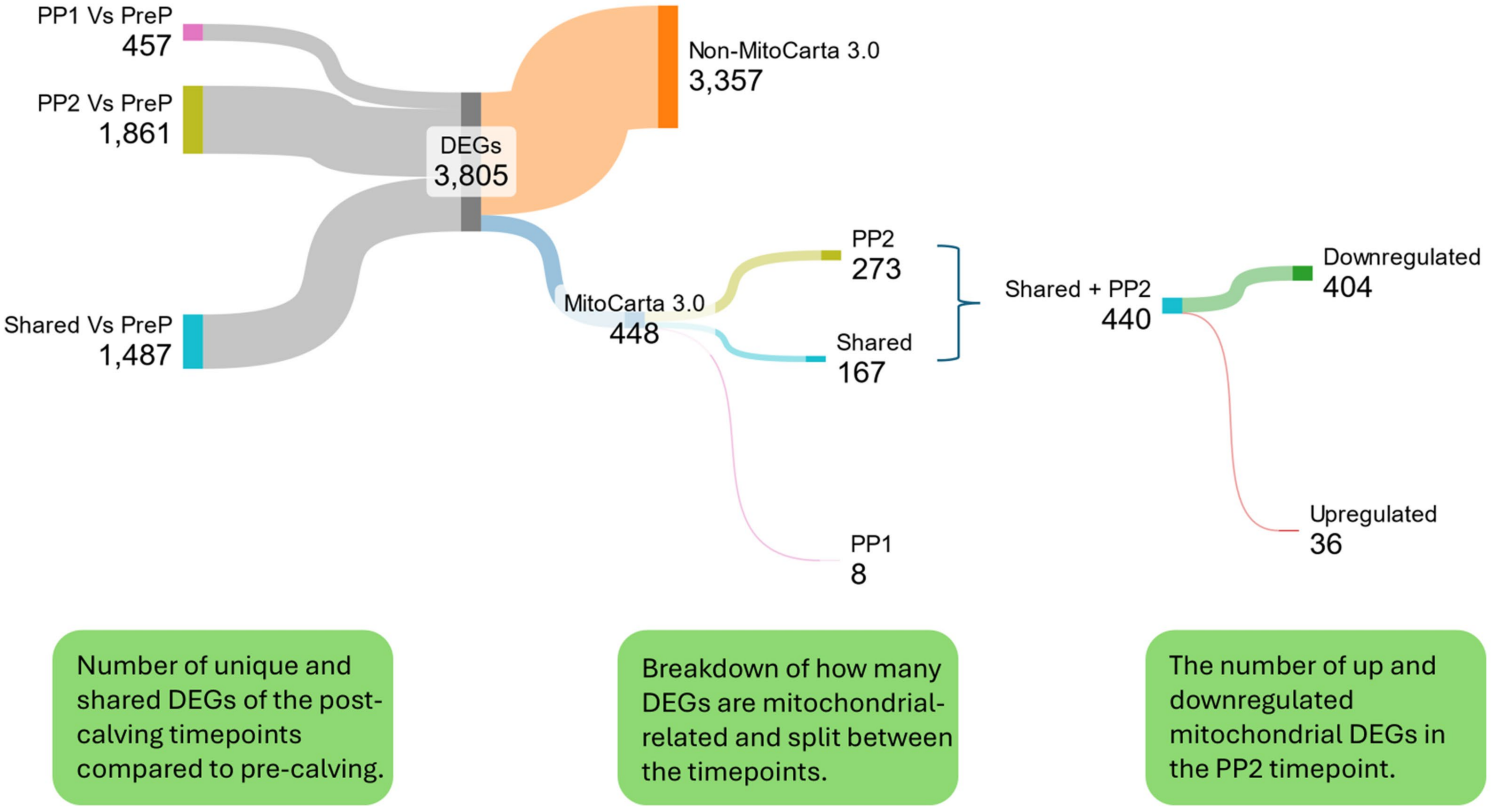
The distribution of DEGs in adipose tissue across the 3 timepoints (pre-calving PreP compared with the two post-calving timepoints PP1 and PP2). DEGs were identified based on a fold change > 1 and a false discovery rate (FDR) < 0.05. A greater number of DEGs were observed in the PP2 vs. PreP comparison (1,861), with 1,487 shared DEGs between the two post-calving timepoints and 457 unique to PP1 vs. PreP. Of the combined 3,805 DEGs, 448 were mitochondrial-related (as identified using the MitoCarta 3.0 database). The vast majority of mitochondrial-related DEGs (440) were observed in the PP2 vs. PreP comparison, with 404 downregulated and 36 upregulated. Graphic made using Sankeymatic.com.

Total RNA extracted from the subcutaneous AT samples at PreP, PP1, and PP2 was sequenced and subjected to quality control as previously described (11) (samples were collected in 2019, flash-frozen and sequenced by Novogene within 3 months; the present analysis revisits those data). Post-sequencing, the gene count matrix was analysed using NetworkAnalyst 3.0, to filter out genes with low transcription abundance and constant values, followed by log2 normalisation of gene counts (15). Principal component analysis (PCA) and 3D PCA analyses were conducted, with edgeR package employed for differential expression analysis (16). Genes exhibiting fold changes > 1 and False Discover Rates (FDRs) < 0.05 were identified as DEGs for further analysis. Pathway enrichment was originally performed using Ingenuity Pathway Analysis (IPA), which relies on a proprietary knowledge base and licence-restricted content. To improve reproducibility and allow independent verification, we repeated the enrichment using only publicly accessible, citable resources [DAVID, STRING-db, WikiPathways/PathVisio and MitoCarta (see below)]. This allows other investigators to re-run the analysis directly from the GEO dataset (GSE159224), review the gene lists, thresholds and multiple-testing corrections, and trace each pathway or term back to publicly curated records. Where IPA features (such as proprietary upstream-regulator predictions), had no direct public equivalent, we used network-based enrichment in STRING-db and community-maintained pathway maps in WikiPathways/PathVisio. These changes preserve the biological conclusions, improve methodological transparency, and removes dependence on private sources, aligning the study with FAIR and open science best practices (see Figures 1, 2).

### Open-source functional enrichment re-analysis of the transcriptomes

The DEGs from both the PP1 and PP2 comparisons were cross-referenced against the MitoCarta 3.0 database (17) (a comprehensive inventory of mammalian mitochondrial genes), using Venny 2.1.^1^ This step was taken to identify DEGs with known mitochondrial function and better isolate mitochondrial changes within the dataset.

Gene Ontology term analysis was conducted as an initial step to provide a broad overview of the biological processes, molecular functions, and cellular components impacted by DEGs in PP2 vs. PreP, using the tools described below. Building on these findings, functional enrichment was employed to categorise DEGs into specific clusters and pathways.

Enrichment analyses of upregulated and downregulated PP2 transcripts were conducted with STRING-db a functional protein association network,^2^ to identify significant biological processes (FDR < 0.05) (18). The resulting GO terms were visualised, and graphs were generated using Matplotlib (version 3.4.3). Downregulated transcripts were grouped into distinct clusters using k-means clustering, and the optimal number of clusters was determined using the elbow method (19). This process was performed in R (20) and identified four clusters of downregulated transcripts in the PP2 vs. PreP comparison with GO terms visualised using Matplotlib (version 3.4.3) (21).

Gene identifiers from the DEGs list were converted to recognised gene symbols using the DAVID Bioinformatics Resources 6.8^3^ conversion tool. Following the conversion, DEGs from the PP2 upregulated and downregulated transcripts were categorised into functional clusters using DAVID, this tool brings together information from Gene Ontology (GO), KEGG pathways, and other curated datasets to help group DEGs into meaningful biological categories based on common features or functions. The counts of DEGs in the top three enrichment categories for each cluster were visualised using Matplotlib (version 3.4.3) (21), which is a Python 2D library, plotting -log10 (FDR) values against the terms with colour intensity reflecting enrichment strength (21). Mitochondrial DEGs from the PP2 vs. PreP dataset were cross-referenced with MitoCarta3.0 to classify genes linked to oxidative phosphorylation (complexes I–V) and uncoupling proteins, using the R package “dplyr” (20).

Genes and pathways within GeneCards (22), were cross-referenced with the DEGs, also via the R studio package “dplyr” (20), bar graphs were generated with Matplotlib (version 3.4.3) (21).

The DEGs in the PP2 vs. PreP comparison were integrated with Wikipathways (23) using PathVisio software (24) with colour-coded annotations to indicate changes in transcript levels. Pathway selection was informed by the biological themes emerging from GO and DAVID enrichment results, specifically those relating to mitochondrial respiration, glycolysis, and inflammatory signalling.

Stage-matched contrasts of dietary oleic acid versus control were also re-analysed from the same transcriptomic dataset (accession number: GSE159224) (11) using the identical pipeline (edgeR, FDR < 0.05). Analyses did not identify additional significant DEGs after correction (Supplementary materials 1–3).

### Lactate and metabolite analysis

Blood samples were collected at PreP, PP1, and PP2 via coccygeal venipuncture using coated collection tubes (K_2_ EDTA) before morning feeding and stored on ice. Samples were then centrifuged at 2,000 x g for 15 min at 4 °C for plasma fraction collection and then stored at −20 °C until further analysis. L-Lactate detection and quantification was performed using the Lactate-Glo Assay (J5022; Promega) following manufacturer’s protocol. Briefly, plasma samples were thawed on ice and diluted (1:20) in 1X PBS. 50 μL of diluted plasma samples were added into the wells of a white 96-well assay plate. 50 μL of freshly prepared Lactate Detection Reagent was added. Plate was placed on a shaker for 60 s to mix and then incubated at room temperature for 60 min protected from light. After incubation, luminescence was recorded using BioTek Synergy H1 plate reader.

Additionally, plasma samples from 22 clinically ketotic (CK) cows and 19 healthy controls (HC) were analysed to investigate lactate correlations with metabolic parameters, which were sourced from a previously conducted randomized clinical trial investigating lipolysis inhibition in clinical ketosis (25). The trial was conducted over a 7-month period (*n* = 1,250), in a commercial Jersey dairy herd of 2,645. Cows were classified as CK primarily on the basis of clinical signs (depressed appetite, reduced rumen fill, and lethargy), and subsequently confirmed if blood *β*-hydroxybutyrate (BHB) concentrations were ≥1.2 mmol/L, in accordance with previously established thresholds and matched with controls (25). The study was approved by the Institutional Animal Care and Use Committee (IACUC) at Michigan State University (AUF: 202100139).

Blood sampling and processing for these CK and control cows followed the same protocol described above. Plasma lactate was measured as described above. Non-esterified fatty acids (NEFA) were quantified using a colorimetric enzymatic assay (HR Series NEFA-HR(2), Wako Diagnostics) following the manufacturer’s protocol. Plasma glucose, total protein, BHB, and triglycerides were measured using a small-scale automated biochemistry analyser (CataChemWell-T, Catachem Inc.).

## Results and discussion

### Early lactation (PP2) coincides with a pronounced downregulation of mitochondrial-related transcripts

Transcriptomics analyses revealed a downregulation of mitochondrial-related genes during the PP2 vs. PreP comparison. Of the 448 mitochondrial-related DEGs identified utilising the MitoCarta 3.0 database, 440 were in PP2 vs. PreP, with 404 of those DEGs downregulated and 36 upregulated. This observed downregulation of DEGs in the PP2 comparison suggests a shift in gene expression for FA oxidation and OXPHOS, which may impair adipose tissue to efficiently mobilise reserves. To explore this further with the transcriptomic data, GO Term analyses were undertaken for both the upregulated and downregulated PP2 vs. PreP DEGs.

### Early lactation (PP2) transcriptomic profiles reveal increased immune and inflammatory activity and reduced bioenergetic function

**Figure 3:**
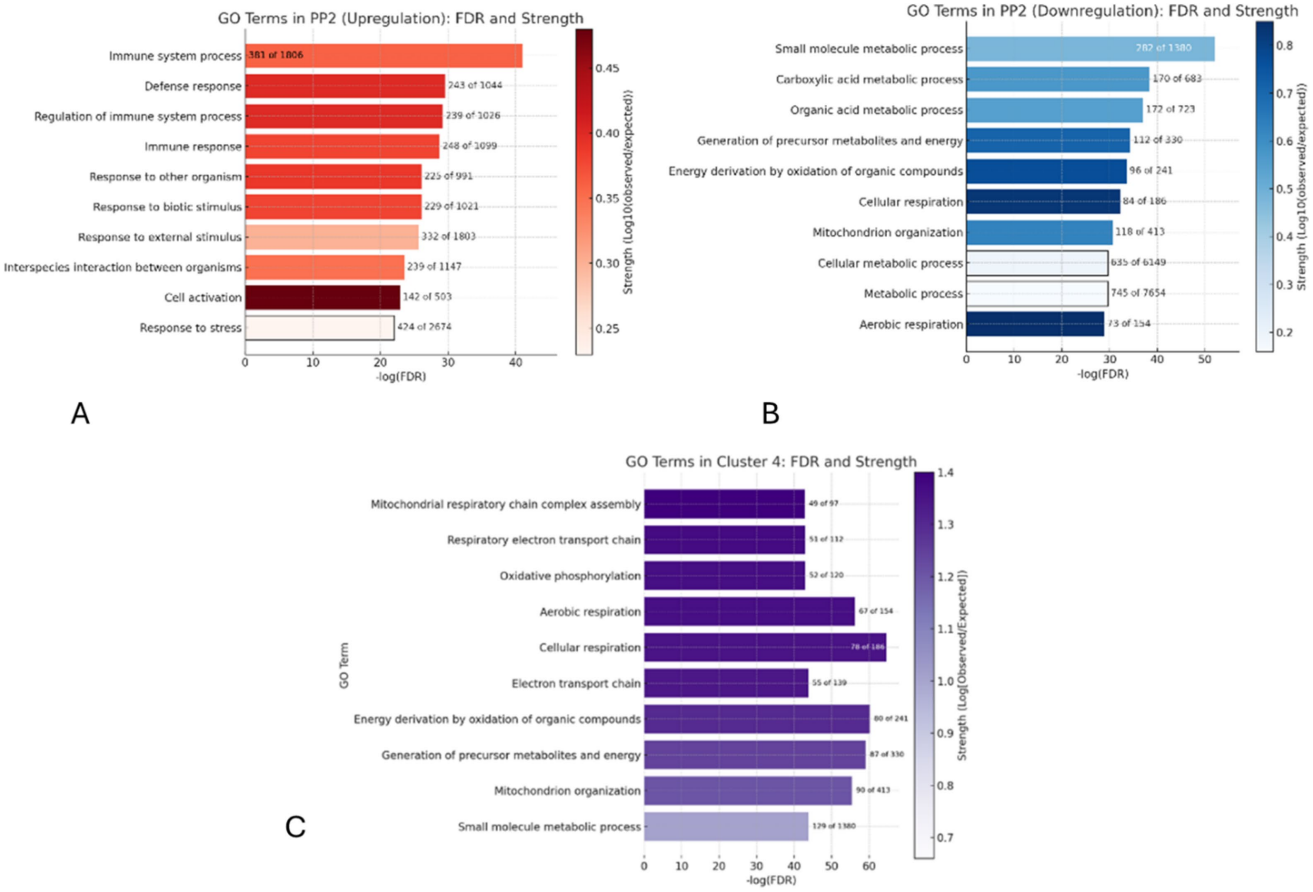
GO terms enriched in PP2 upregulation, ranked by FDR significance on the x-axis and enrichment strength (colour). **(A)** Bars show the number of DEGs per term. Immune processes dominate, highlighting a strong immune response in PP2. **(B)** GO terms enriched in PP2 downregulation, Metabolic and mitochondrial processes are changed, suggesting an alteration of energy metabolism in PP2. **(C)** GO terms enriched in PP2 downregulation (GO cluster 4), Mitochondrial and oxidative processes dominate, suggesting a strong focus on energy metabolism and respiration.

GO term analysis of the upregulated DEGs in PP2 vs. PreP revealed that the most prominent process was the “Immune system process” with 381 out of 1,806 upregulated genes (FDR: 8.83e-42), followed by “Defense response” and “Regulation of immune system process” (Figure 3). The main GO terms associated with downregulated transcripts in PP2 were “small molecule metabolic processes,” with 282 of the 1,380 downregulated genes (FDR: 6.78e-53). Other affected processes include “Carboxylic acid metabolic process,” “Mitochondrion organisation,” and “Organic acid metabolic process” (Figure 3B).

Using K-means clustering, with the optimal number of clusters determined using the elbow method (19) four distinct clusters were identified in downregulated PP2 transcripts. GO Cluster 4 was the largest, comprising 444 out of a total of 1,463 DEGs, containing GO Terms related to mitochondrial function. The GO Terms “Mitochondrial respiratory chain complex assembly” and “Respiratory electron transport chain” had the greatest number of DEGs showing significant enrichment (FDR: 4.8e-97 and 5.9e-132, respectively). This cluster’s focus on energy production is further evidenced by the inclusion of terms like “Oxidative phosphorylation” and “Aerobic respiration” (Figure 3C). Briefly, the remaining clusters highlighted processes including protein catabolism (Cluster 1), lipid biosynthesis (Cluster 2), and cellular response mechanisms (Cluster 3) (detailed GO term enrichments provided in Supplementary Figures 1–3).

### Functional clustering of PP2 transcripts highlights changes in mitochondrial and immune activity

Functional enrichment (FE) was utilised to extend the GO term analysis by identifying biological pathways and regulatory networks. Functional clustering in PP2 vs. PreP showed that upregulated transcripts were mostly in immune-related categories (Supplementary Figure 4). FE Cluster 1 was most enriched in immune activity, with the highest DEG counts (83) (P Val. 2.5E-22) in the “Immune response” category. FE Cluster 2 had significant counts in “Calcium ion binding” (99) (P Val. 9.0E-10) (Supplementary Figure 4).

Clustering of downregulated transcripts in PP2 showed the enrichment of mitochondrial activity. FE Cluster 1 showed a count of 307, and P Val of 4.0E-149 for cellular component “Mitochondrion,” FE Cluster 2 revealed a count of 78 and P Val, of 8.0E-47 for the KEGG Pathway “Oxidative Phosphorylation” (Supplementary Figure 5).

The GO term analysis and functional enrichment revealed two main themes: immune activation and mitochondrial dysfunction. Similar patterns have been described in human and rodent models of obesity and diabetes, where mitochondrial stress is linked to immune activation, creating a damaging feedback (26). Similarly, a review of periparturient lipolysis in cows describes how intense lipolysis triggers mitochondrial oxidative stress, which perpetuates inflammation in a vicious cycle (6). To gain deeper insight into the mitochondrial role in these processes in bovine adipose tissue, specific pathways were analysed, including mitophagy, apoptosis, oxidative stress, and calcium signalling.

### Exploration of specific pathways related to metabolic stress, pro-inflammatory signalling and mitochondrial dysfunction

Analyses of specific pathways via GeneCards, based on the GO Terms and Functional Enrichment results, were undertaken to identify relevant biological processes beyond broad functional classifications.

Immune activation is evident from the significant upregulation of acute-phase proteins, notably haptoglobin (*HP*) and *IL-6* (Supplementary Figure 6a), with adjusted *p*-values of 7.19 × 10^−12^ and 5.38 × 10^−7^, respectively. These transcripts highlight a strong pro-inflammatory state (6), potentially geared c protecting against metabolic and microbial challenges. Conversely, the downregulation of *RNASE2, CAMK2B*, and *PRLR* may indicate a reprioritisation of immune-regulatory pathways, aligning with the elevated inflammatory state (27–29) (the full names of selected DEGs are provided in Supplementary Table 1). Overall, these changes denote an acute-phase immune response in adipose tissue (6).

The upregulation of oxidative stress-related genes, including *IL-6, TREM2, IGFBP3*, and *HMOX1*, in the PP2 vs. PreP dataset (Supplementary Figure 6b) suggests an increased inflammatory and oxidative response; in contrast, *GSTT1*, *SGK2*, *ALB*, and *G6PD*, genes linked to antioxidant defence and redox regulation were downregulated, indicating potential disruptions in oxidative stress management and cellular homeostasis (30–32). These changes indicate a metabolic shift in which stress-response activation is coupled with reduced antioxidant capacity, potentially affecting overall cellular resilience. Lipopolysaccharide-binding protein (*LBP*) is also upregulated, indicating an active defence mechanism against endotoxins (33). Meanwhile, the downregulation of *GDF5*, alongside the upregulation of transcripts such as *CXCR4* and *LITAF*, suggests a reprogramming of cytokine and chemokine signalling pathways associated with the LPS response cluster (34) (Supplementary Figure 6d).

Mitochondrial quality control denotes the pathways of repair, generation and clearance of mitochondria to sustain cellular energy dynamics. This process was evident from prominent changes in mitophagy-related transcripts; *SPATA18* was highly upregulated, pointing to increased mitophagy (35). Other mitophagy-related changes included *SRC* and *VPS13C* upregulation, while *MFN2, PRKN (PARKIN), PINK1, OPTN, VDAC1,* and *SLC25A4* were downregulated, indicating significant alterations in mitochondrial quality control and turnover (36) (Supplementary Figure 6c). Several components of mitochondrial calcium ion transport showed reduced expression, including *VDAC1-3*, *LETM1*, and the *MCU* complex, which are essential for mitochondrial ion exchange (Supplementary Figure 6f) (37). Concurrently, *MCUB* was upregulated, suggesting a potential shift in calcium transport dynamics (37). Transcripts linked to the mitochondrial apoptosis pathway showed increased expression, including *CASP8, MCL1*, and *BAX*, suggesting enhanced apoptotic signalling (38) (Supplementary Figures 6e,f).

Collectively, the shift towards immune activation, oxidative stress, and altered mitochondrial function, suggests that AT may be undergoing metabolic reprogramming postpartum.

### Metabolic reprogramming

The downregulation of OXPHOS components across Complexes I to IV during PP2 indicates a significant metabolic shift (Supplementary Figure 7), supporting an adaptive strategy to altered energetic demands (39). The upregulation of *UCP2* alongside OXPHOS downregulation suggests an effort to balance energy production and mitigate oxidative stress (40).

Cellular metabolic reprogramming (a phenomenon widely described as the Warburg effect, in tumour biology), involves shifting from primarily oxidative phosphorylation to elevated glycolysis, even in the presence of adequate oxygen (41). Studies show that non-tumour cells, including adipocytes, can adopt this approach under stress to meet urgent energy demands and generate essential metabolic intermediates (42). In postpartum dairy cows, such a physiological transition may help adipose tissue cope with sudden metabolic pressures linked to milk production while reducing ROS produced by fatty acid oxidation. However, prolonged reliance on glycolysis could diminish overall oxidative capacity, intensify inflammation, and disrupt tissue homeostasis; changes that match the strong immune signals and reduced mitochondrial transcript abundance seen in the data. This prompted a further exploration, focusing on metabolic reprogramming pathways and transcripts.

STRING-db network analysis (which predicts protein–protein interaction networks to reveal enriched biological pathways) of Pyruvate Carboxylase (*PC*; an enzyme that aids in replenishment of TCA cycle) portrays increased glycolytic flux, with *PKLR, LDHA*, *ME2*, and *PKM* upregulated (involved in pyruvate metabolism and glycolytic throughput), while reduced substrate entry into the TCA cycle is suggested by the downregulation of *PDHA1, GOT1, GOT2, ALDOA*, and *PC* (5, 43) (Supplementary Figure 8). GeneCards pathway analysis reinforces metabolic reprogramming, showing upregulation of *HK3, LDHA*, and *PKM*, consistent with enhanced glycolysis (Supplementary Figure 9). Meanwhile, the suppression of *PDHA1, ACO2*, and *SLC16A1* reflects impaired mitochondrial substrate utilisation, and downregulation of *G6PD, HK2*, and *TKT* suggests reduced pentose phosphate pathway activity, potentially affecting redox balance (5). The downregulation of *ACLY* and *FASN* indicates a shift away from lipogenesis, indicating changes in energy partitioning (5, 44). Interestingly, *MCT4* (*SLC16A3*) was upregulated, while *MCT1 (SLC16A1)* was downregulated, suggesting a shift toward lactate export.

**Figure 4:**
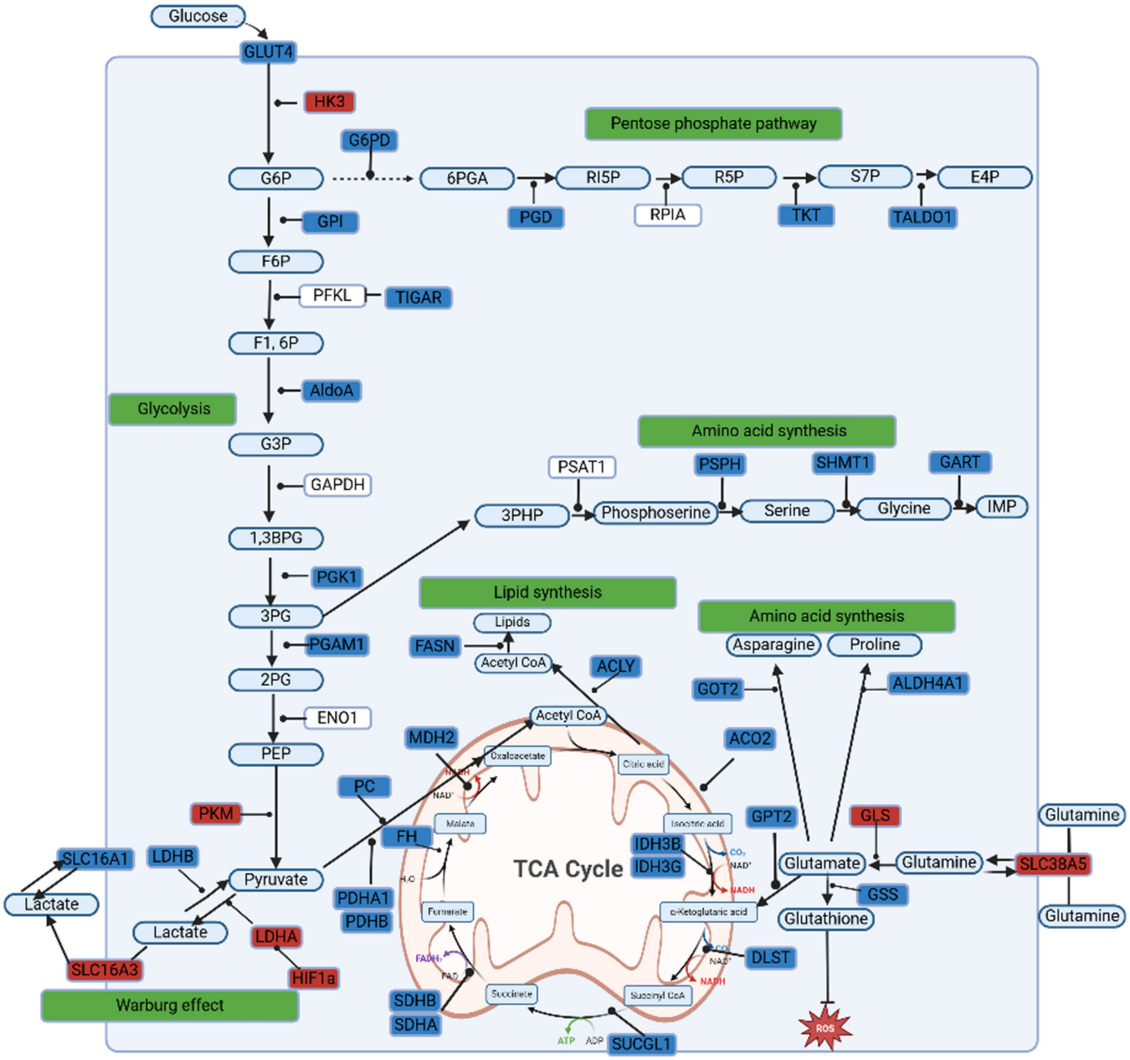
Visualisation of metabolic reprogramming pathways in bovine adipose tissue. DEGs in red are upregulated, blue are downregulated. In combination, these shifts are consistent with adipose tissue metabolic reprogramming. Adapted from WikiPathways (23).

Enhanced glycolysis is evident (*HK3, PKM, LDHA*) with concurrent reduced OXPHOS (*PDHA1, ACO2, IDH3G, SDHB*) pointing to lower mitochondrial functionality (5) (Figure 4). Lipogenesis is decreased (*FASN, ACLY*), but there is an upregulation in DEGs associated with lactate export (*LDHA, SLC16A3*) (5). Increased *HIF1*α denotes hypoxia. However, although we assume this is metabolic reprogramming reminiscent of the aforementioned Warburg effect (41), postpartum adipose tissue is not driving uncontrolled proliferation or building block synthesis. Instead, these changes may be supporting the heightened energy demands of early lactation, producing and exporting lactate, managing redox balance, and adjusting substrate usage.

A similar metabolic reprogramming profile occurs in physiological and pathological settings such as human cancer cachexia, where immune activation and TLR4 signalling drive lipolysis, tissue remodelling, and thermogenic activation of white AT (42). Muscle loss in dairy cows also fits with an energy deficit, a proteomic study in early-lactation Holstein muscle reports a similar suppression of TCA cycle proteins and induction of enzymes for glycolysis, fatty acid breakdown, and lactate metabolism (45).

The downregulation of TCA cycle enzymes (e.g., *MDH2, IDH3B*) and upregulation of glutaminolysis genes (*GLS, SLC38A1*) that we see indicate a shift toward glutamine metabolism (46) (Figure 4). This process can increase muscle protein breakdown, generating lactate and alanine that replenish the TCA cycle via substrate-level phosphorylation (46). Elevated HIF1α also signifies a hypoxic response, pushing cells from OXPHOS toward glycolysis and lactate production (5).

*UCP2* and *SLC38A1* upregulation corroborates the idea of a metabolic shift, by facilitating glutamine uptake and usage when glucose availability is restricted (40). *UCP2* reduces mitochondrial pyruvate oxidation, lowers glucose oxidation, and boosts glutamine metabolism, a pattern that may underlie disease-related metabolic adaptations (40). Its co-expression with *SLC38A1* heightens metabolic flexibility, and in immune cells, *UCP2* is pivotal for glutamine oxidation and regulation of ROS (40). Although the transcript-level changes are consistent with a potential shift toward glutamine metabolism and a Warburg-like metabolic profile, these interpretations remain speculative without direct flux measurements or additional functional assays. While the data hint at metabolic adaptations involving glutaminolysis and hypoxia-induced reprogramming, further experimental evidence is required to confirm whether these changes genuinely reflect Warburg-like metabolism in postpartum adipose tissue.

### Transcriptomic insights into the potential role of lactate in postpartum metabolism

The AT continuously adjusts between OXPHOS and glycolysis based on oxygen levels and energy demands (47). While OXPHOS, driven by fatty acid oxidation, dominates under normal conditions, acute energy stress or hypoxia shifts metabolism toward glycolysis for rapid ATP production (41). Recent studies have also highlighted lactate, as a stimulant for OXPHOS, a regulator of redox balance, and a key player in maintaining mitochondrial homeostasis (9), indicating that the ability, flux and rate of lactate export are critical. Our transcriptomic analysis shows this shift may be happening postpartum in adipose tissue, with OXPHOS transcripts downregulated and glycolytic pathways upregulated. This abrupt energy demand disrupts redox balance, altering the NADH/NAD^+^ ratio and effecting metabolic reprogramming, similar to other high-demand states such as cancer, immune activation, and exercise (9, 41). Rather than supporting local oxidative phosphorylation or lipid storage, AT may be shifting towards lactate export. Lactate is potentially oxidised in local tissues, supplying the liver for gluconeogenesis precursors or the mammary gland for milk production (48). Simultaneously, the upregulation of glutamine transporter *SLC38A5* and glutaminase (GLS) suggests glutamine metabolism may provide alternative TCA cycle intermediates, further supporting metabolic rerouting under mitochondrial downregulation (5).

### The evolving role of lactate in adipose tissue metabolism

Our findings support the evolving view that AT is no longer seen solely as an energy store, but rather a dynamic organ that has endocrine function, along with regulating redox and inflammatory states (49). Lactate is now considered to be a signalling molecule that bridges OXPHOS and glycolysis (9). Lactate is integral to adipocyte metabolism, extending beyond glycolysis to control glucose balance (even under insulin resistance), redox homeostasis, and immune function (9). Lactate also has a role in stabilising HIF-1α, and influences macrophage polarisation and associated inflammation (50). Lactate production increases substantially in adipocytes under hypoxia, reflecting a metabolic shift that redirects glucose from oxidative metabolism toward glycolysis, rewires glutamine utilisation, and alters fatty acid synthesis (51). Furthermore, lactate flux in adipocytes was shown to coordinate a proinflammatory cascade, drive insulin resistance and explain lactate’s pivotal role in obesity-linked metabolic dysfunction (52). It has been demonstrated that lactating cows dynamically adjust hepatic lactate uptake based on substrate availability (53). Considering the intense energy demands of early lactation and lactate’s increasingly recognised role in adipose tissue dynamics (spanning redox balance, inflammatory regulation, and insulin resistance), further investigation into this shift toward glycolysis and lactate-driven metabolism may offer novel insights into metabolic health in postpartum dairy cows.

### Plasma lactate decreases in early lactation (PP2) relationship with metabolic markers in control and ketosis

**Figure 5:**
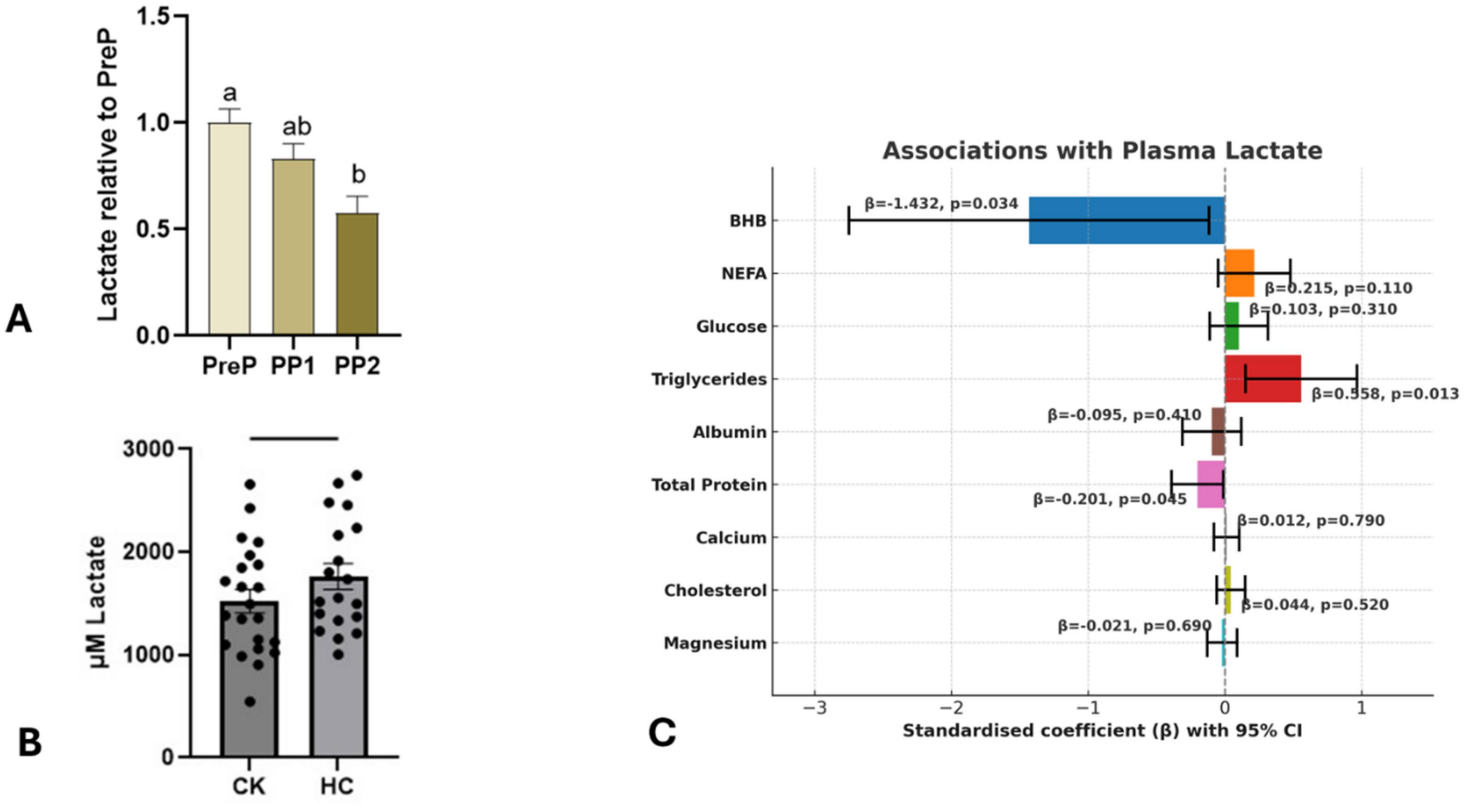
**(A)** Lactate concentrations were measured in cows across the three biopsy periods: PP1, PP2, and PreP. Least squares means (LS Means) estimates for lactate concentrations, relative to the PreP period, are shown for each biopsy period. The error bars represent the 95% confidence intervals for the LS Means estimates. PP2 showed lower lactate concentrations than both PreP and PP1, whereas PP1 did not differ from PreP. Tukey–Kramer pairwise comparisons confirmed significant differences for PP2 vs. PP1 (*p* = 0.036) and PP2 vs. PreP (*p* = 0.0007), with no significant difference between PP1 and PreP (*p* = 0.15). **(B)** Comparison of plasma lactate concentrations between control (Healthy Cows HC) and Ketotic (Clinically Ketotic, CK) Each point represents an individual cow. The bars represent the mean lactate concentration, and the error bars denote the standard error of the mean (SEM). An initial significant difference in lactate concentration was not observed between the groups but was close at *p* < 0.06. **(C)** Standardised coefficients between plasma lactate concentrations and various metabolic parameters in both control and ketotic cows. The parameters analysed include albumin, BHB, calcium, cholesterol, glucose, magnesium, NEFA, total protein (TP) and triglycerides. Plasma lactate was significantly associated with BHB (coefficient = −1.4319, *p* = 0.03396).

Plasma lactate concentrations were measured across the three biopsy periods; PreP (11 ± 3.6 days before calving), PP1 (6 ± 1 day post-calving), and PP2 (13 ± 1.4 days post-calving) (Figure 5). Concentrations were significantly lower at PP2 than at both PreP (*p* = 0.0007) and PP1 (*p* = 0.036), whereas PP1 did not differ from PreP (*p* = 0.15) (see Supplementary Table 2 for LS-means estimates, confidence intervals, and adjusted pairwise comparisons). The decline in PP2 may arise from several factors, such as reduced adipose-derived lactate production or altered systemic clearance, potentially driven by increased mammary uptake, enhanced hepatic gluconeogenesis, or greater oxidative metabolism in peripheral tissues. Although a multitude of factors may be causal, it coincides with intense change in mitochondrial function, energy redirection and tissue redox levels.

Like lactate, ketones also serve as alternative metabolic fuels, and ketone metabolism is closely linked to mitochondrial function, insulin resistance, hypoxia, and oxidative stress (10). Excessive ketone accumulation due to prolonged negative energy balance, is associated with various metabolic and health issues in dairy cows (1). We examined plasma lactate concentrations in control and clinically ketotic cows to better understand their dynamics.

Plasma lactate concentrations were compared between clinically healthy (HC, *n* = 19) and ketotic (CK, *n* = 22) cows to assess metabolic differences in clinical ketosis (Figure 5B). While no significant difference was observed (*p* = 0.06), a trend toward lower lactate levels in ketotic cows was noted.

In controls, lactate positively correlates with NEFA and triglycerides (Supplementary Table 3), linking higher lactate to enhanced fat mobilisation. Whether this reflects a direct role in lipid mobilisation or an adaptive metabolic response is not clear. However, in ketotic cows, lactate instead shows a negative relationship with total protein (Supplementary Table 4), and no significant ties to NEFA or triglycerides. In the combined regression analysis (Figure 5C), the inverse association between plasma lactate and BHB, could point to a compensatory relationship between these two metabolites. Lactate has also been shown to suppress adipose lipolysis via the G-protein–coupled receptor GPR81, lowering cAMP and limiting hormone-sensitive lipase activity (54). Therefore, the lower lactate observed in ketotic cows may reflect loss of this anti-lipolytic brake, contributing to the elevated NEFA. Ketogenesis plays an important role in regenerating NAD^+^ during fatty acid oxidation, providing an alternative mechanism to manage redox stress when lactate metabolism alone is insufficient (5). This shift suggests that under more extreme metabolic stress, ketones may take over as the dominant energy source and redox buffer, reflecting a system under prolonged metabolic strain.

### Implications for diagnosis and therapy

Our finding of heightened immune and inflammatory gene expression, together with the observed lactate–ketone toggle, is supported by a study that shows changes in IL-6 and lactate/BHB dynamics can precede ketosis (55). A greater understanding of the mitochondrion’s role in inflammatory and immune function during lipolysis, may lead to novel interventions to prevent and treat ketosis and elucidate its role in the transition cow disease process. Further longitudinal studies would aid clarification but involving lactate and inflammatory/immune markers could already provide a more informative toolset for ketosis diagnosis and management.

### Limitations

As with any transcriptomic study, these data do not support conclusions of functional outcomes but provide additional evidence that will need to be validated with further targeted functional experiments.

It is possible some of the transcriptomic shifts reported in PP2 may reflect differences in cellular composition of subcutaneous AT, rather than intrinsic transcriptional changes. We did not carry out cell-type specific resolution or immune phenotyping in this study, so that distinction cannot be made here. Future work using approaches such as cell deconvolution, flow cytometry, or single-cell and single-nucleus RNA sequencing will be needed to separate changes in cell abundance from reprogramming within existing cell types. The study was also limited by sample size, which meant we were unable to fully assess the oleic acid treatment effect.

## Conclusion

Our study provides initial transcriptomic and biochemical evidence that metabolic reprogramming occurs at the transcription level in subcutaneous AT of postpartum dairy cows, characterised by a pronounced downregulation of mitochondrial transcripts, a shift away from OXPHOS, and a concurrent activation of immune and stress-response pathways. Our data support the concept that adipose tissue is not merely a passive energy store but plays an active role in regulating metabolic and inflammatory processes during the postpartum energy demand (4).

The placement of lactate and ketones as reciprocal redox regulators and energy sources, aligns with the evolving view of ketone metabolism. This challenges the perception of ketosis as strictly pathological, and proposes that ketones may function as adaptive metabolic signals under certain conditions (56, 57).

We provide evidence and highlight that metabolic health in dairy cows cannot be fully understood solely through traditional markers like ketone bodies. Instead, a better understanding of mitochondrial adaptations, immune-metabolic interactions, and tissue-specific energy partitioning, is required, to refine the understanding of periparturient metabolic disorders. Future research should focus on whether these mitochondrial and metabolic shifts can be modulated to improve metabolic resilience (14), potentially reducing the risk of disease while maintaining optimal production efficiency.

## Data Availability

The datasets presented in this study can be found in online repositories. The names of the repository/repositories and accession number(s) can be found at: https://www.ncbi.nlm.nih.gov/geo/query/acc.cgi?acc=GSE159224.

## Footnotes

^1^ https://bioinfogp.cnb.csic.es/tools/venny/index.html

^2^ https://string-db.org/

^3^ https://davidbioinformatics.nih.gov/

## Supplementary material

SUPPLEMENTARY FIGURE 1 The top 10 enriched GO terms were ranked by False Discovery Rate (FDR) for Cluster 1 using STRING-db. The bar length represents the-log10(FDR) value, and the colour intensity indicates the enrichment strength (log10(observed/expected)).

SUPPLEMENTARY FIGURE 2 The top 10 enriched GO terms were ranked by False Discovery Rate (FDR)for Cluster 2 using STRING-db. The bar length represents the-log10(FDR) value, and the colour intensity indicates the enrichment strength (log10(observed/expected)).

SUPPLEMENTARY FIGURE 3 The top 10 enriched GO terms were ranked by False Discovery Rate (FDR) for Cluster 3 using String-db. The bar length represents the -log10(FDR) value, and the colour intensity indicates the enrichment strength (log10(observed/expected)).

SUPPLEMENTARY FIGURE 4 Functional enrichment of PP2 upregulated transcripts, showing DEG counts across the top three clusters. Bars represent transcript counts per enrichment category, with colours distinguishing clusters. FE Cluster 1 (purple) is enriched in immune-related processes, FE Cluster 2 (teal) in calcium and growth factor binding, and FE Cluster 3 (yellow) in SH2 domain-associated signalling.

SUPPLEMENTARY FIGURE 5 Functional enrichment of PP2 downregulated transcripts FE Cluster 1 (purple) is enriched in mitochondrial and oxidative metabolism, FE Cluster 2 (teal) in electron transport and neurodegenerative pathways, and FE Cluster 3 (yellow) in TCA cycle-associated processes.

SUPPLEMENTARY FIGURE 6 Upregulated (red) and downregulated (blue) transcripts in PP2 vs. PreP were cross-referenced with pathways from GeneCards. **(a)** Immune Response. **(b)** Oxidative Stress. **(c)** Mitophagy. **(d)** LPS-related. **(e)** Apoptosis regulation. **(f)** Calcium-related mitochondrial genes.

SUPPLEMENTARY FIGURE 7 The electron transport system (complexes I–V) is depicted with associated mitochondrial- and nuclear-encoded genes. Downregulated transcripts (blue) indicate widespread suppression of nuclear-encoded OXPHOS components, and Complex V, while UCP2 (red) highlights an upregulation in uncoupling activity.

SUPPLEMENTARY FIGURE 8 STRING-db network visualisation depicting protein-protein interactions among key metabolic enzymes linked to pyruvate carboxylase (PC). Red nodes represent upregulated proteins, while blue nodes indicate downregulated proteins. Connectivity between nodes represents functional and regulatory interactions.

SUPPLEMENTARY FIGURE 9 All upregulated, and the top 10 downregulated transcripts associated with metabolic reprogramming. Red bars indicate upregulated transcripts, while blue bars represent downregulated transcripts, with log fold change (logFC) values shown on the x-axis.

SUPPLEMENTARY TABLE 2 Lactate concentrations were measured in cows across the three biopsy periods: PP1, PP2, and PreP. Least squares means (LS Means) estimates for lactate concentrations, relative to the PreP period, are shown for each biopsy period. The error bars represent the 95% confidence intervals for the LS Means estimates. P2 had lower lactate concentrations than both PreP and PP1, whereas PP1 did not differ from PreP. Tukey–Kramer pairwise comparisons confirmed significant differences for PP2 vs. PreP (*p* = 0.0007) and PP2 vs. PP1 (p = 0.036), with no significant difference between PP1 and PreP (*p* = 0.15). Least-squares means are relative to the PreP period. Significant differences (*p* < 0.05) are shown in bold.

SUPPLEMENTARY TABLE 3 Correlations between plasma lactate concentrations and various metabolic parameters in control cows. The parameters analysed include albumin, BHB, calcium, cholesterol, glucose, magnesium, NEFA, total protein (TP) and triglycerides. Plasma lactate was significantly correlated with both triglycerides (r = 0.5579, *p* = 0.0131) and NEFA (r = 0.5702, *p* = 0.0108).

SUPPLEMENTARY TABLE 4 Correlations between plasma lactate concentrations and various metabolic parameters in ketotic cows. In this ketotic group, lactate shows a significant negative correlation with total protein (r = –0.4345, *p* = 0.038) but no meaningful correlation with triglycerides or NEFA. This pattern differs from controls, where lactate was notably linked to lipid mobilisation markers (NEFA and triglycerides).

## References

[1] Kang D Lungu SE Danso F Dzou CF Chen Y Zheng X . Animal health and nutrition: metabolic disorders in cattle and improvement strategies. Front Vet Sci. (2025) 12. doi: 10.3389/fvets.2025.1470391, PMID: 40201075 PMC11977490

[2] Britt JH Cushman RA Dechow CD Dobson H Humblot P Hutjens MF . Review: perspective on high-performing dairy cows and herds. Animal. (2021) 15:100298. doi: 10.1016/j.animal.2021.100298, PMID: 34266782

[3] Martins LF Wasson DE Hristov AN. Feeding dairy cows for improved metabolism and health. Anim Front. (2022) 12:29–36. doi: 10.1093/af/vfac059, PMID: 36268175 PMC9564990

[4] Contreras GA Strieder-Barboza C Raphael W. Adipose tissue lipolysis and remodeling during the transition period of dairy cows. J Anim Sci Biotechnol. (2017) 8:41. doi: 10.1186/s40104-017-0174-4, PMID: 28484594 PMC5420123

[5] Chandel NS. Navigating metabolism. CSH Press. (2015). Available online at: https://library.wur.nl/WebQuery/titel/2078973 (Accessed February 4, 2025)

[6] Zachut M Contreras GA. Symposium review: mechanistic insights into adipose tissue inflammation and oxidative stress in periparturient dairy cows*. J Dairy Sci. (2022) 105:3670–86. doi: 10.3168/jds.2021-21225, PMID: 35151484

[7] Qiao K Jiang R Contreras GA Xie L Pascottini OB Opsomer G . The complex interplay of insulin resistance and metabolic inflammation in transition dairy cows. Animals. (2024) 14:832. doi: 10.3390/ani14060832, PMID: 38539930 PMC10967290

[8] De Koster J Strieder-Barboza C de Souza J Lock AL Contreras GA. Short communication: effects of body fat mobilization on macrophage infiltration in adipose tissue of early lactation dairy cows. J Dairy Sci. (2018) 101:7608–13. doi: 10.3168/jds.2017-14318, PMID: 29885887

[9] Brooks GA. The science and translation of lactate shuttle theory. Cell Metab. (2018) 27:757–85. doi: 10.1016/j.cmet.2018.03.008, PMID: 29617642

[10] Puchalska P Crawford PA. Multi-dimensional roles of ketone bodies in fuel metabolism, signaling, and therapeutics. Cell Metab. (2017) 25:262–84. doi: 10.1016/j.cmet.2016.12.022, PMID: 28178565 PMC5313038

[11] Salcedo-Tacuma D Parales-Giron J Prom C Chirivi M Laguna J Lock AL . Transcriptomic profiling of adipose tissue inflammation, remodeling, and lipid metabolism in periparturient dairy cows (*Bos taurus*). BMC Genomics. (2020) 21:824. doi: 10.1186/s12864-020-07235-0, PMID: 33228532 PMC7686742

[12] JH (H) v d K Gross JJ Gerber V Bruckmaier RM. Disturbed bovine mitochondrial lipid metabolism: a review. Vet Q. (2017) 37:262–73. doi: 10.1080/01652176.2017.1354561, PMID: 28712316

[13] Chirivi M Rendon CJ Myers MN Prom CM Roy S Sen A . Lipopolysaccharide induces lipolysis and insulin resistance in adipose tissue from dairy cows. J Dairy Sci. (2022) 105:842–55. doi: 10.3168/jds.2021-20855, PMID: 34696909

[14] Abou-Rjeileh U Neto J d S Chirivi M O’Boyle N Salcedo D Prom C . Oleic acid abomasal infusion limits lipolysis and improves insulin sensitivity in adipose tissue from periparturient dairy cows. J Dairy Sci. (2023) 106:4306–23. doi: 10.3168/jds.2022-22402 37105874

[15] Zhou G Soufan O Ewald J Hancock REW Basu N Xia J. NetworkAnalyst 3.0: a visual analytics platform for comprehensive gene expression profiling and meta-analysis. Nucleic Acids Res. (2019) 47:W234–41. doi: 10.1093/nar/gkz240, PMID: 30931480 PMC6602507

[16] Robinson MD McCarthy DJ Smyth GK. edgeR: a bioconductor package for differential expression analysis of digital gene expression data. Bioinformatics. (2010) 26:139–40. doi: 10.1093/bioinformatics/btp616, PMID: 19910308 PMC2796818

[17] Rath S Sharma R Gupta R Ast T Chan C Durham TJ . MitoCarta3.0: an updated mitochondrial proteome now with sub-organelle localization and pathway annotations. Nucleic Acids Res. (2020) 49:D1541–7. doi: 10.1093/nar/gkaa1011, PMID: 33174596 PMC7778944

[18] Szklarczyk D Gable AL Nastou KC Lyon D Kirsch R Pyysalo S . The STRING database in 2021: customizable protein-protein networks, and functional characterization of user-uploaded gene/measurement sets. Nucleic Acids Res. (2021) 49:D605–12. doi: 10.1093/nar/gkaa1074, PMID: 33237311 PMC7779004

[19] Kassambara A. Practical guide to cluster analysis in R: Unsupervised machine learning /. (2017)

[20] R: The R Project for Statistical Computing. (2023). Available online at: https://www.r-project.org/ (Accessed June 28, 2023)

[21] Hunter JD. Matplotlib: a 2D graphics environment. Comput Sci Eng. (2007) 9:90–5. doi: 10.1109/MCSE.2007.55

[22] GeneCards. Human genes | gene database | gene search. Available online at: https://www.genecards.org/ (Accessed December 11, 2023)

[23] WikiPathways. Available online at: https://www.wikipathways.org/ [Accessed June 28, 2023]

[24] PathVisio. PathVisio biological pathway editor. Pathvisio.Github.io. Available online at: https://pathvisio.github.io//pathvisio.github.io/ [Accessed June 28, 2023]

[25] Chirivi M Cortes-Beltran D Munsterman A O’Connor A Contreras GA. Lipolysis inhibition as a treatment of clinical ketosis in dairy cows: a randomized clinical trial. J Dairy Sci. (2023) 106:9514–31. doi: 10.3168/jds.2023-23409 37678786

[26] Chattopadhyay M Khemka VK Chatterjee G Ganguly A Mukhopadhyay S Chakrabarti S. Enhanced ROS production and oxidative damage in subcutaneous white adipose tissue mitochondria in obese and type 2 diabetes subjects. Mol Cell Biochem. (2015) 399:95–103. doi: 10.1007/s11010-014-2236-7, PMID: 25312902

[27] Boix E Acquati F Leonidas D Pulido D. Editorial: role of ribonucleases in immune response regulation during infection and Cancer. Front Immunol. (2020) 11:236. doi: 10.3389/fimmu.2020.00236, PMID: 32140154 PMC7042197

[28] Yu-Lee L-Y. Prolactin modulation of immune and inflammatory responses. Recent Prog Horm Res. (2002) 57:435–55. doi: 10.1210/rp.57.1.435, PMID: 12017556

[29] Nicole O Pacary E. CaMKIIβ in neuronal development and plasticity: an emerging candidate in brain diseases. Int J Mol Sci. (2020) 21:7272. doi: 10.3390/ijms21197272, PMID: 33019657 PMC7582470

[30] Johnson MA Firth SM. IGFBP-3: a cell fate pivot in cancer and disease. Growth Hormon IGF Res. (2014) 24:164–73. doi: 10.1016/j.ghir.2014.04.007, PMID: 24953254

[31] Ayer A Zarjou A Agarwal A Stocker R. Heme Oxygenases in cardiovascular health and disease. Physiol Rev. (2016) 96:1449–508. doi: 10.1152/physrev.00003.2016, PMID: 27604527 PMC5504454

[32] Sitar M Aydin S Cakatay U. Human serum albumin and its relation with oxidative stress. Clin Lab. (2013) 59:945–52. doi: 10.7754/Clin.Lab.2012.121115 24273915

[33] Bannerman DD Paape MJ Hare WR Sohn EJ. Increased levels of LPS-binding protein in bovine blood and Milk following bacterial lipopolysaccharide challenge. J Dairy Sci. (2003) 86:3128–37. doi: 10.3168/jds.S0022-0302(03)73914-9, PMID: 14594231

[34] Triantafilou M Triantafilou K. Lipopolysaccharide recognition: CD14, TLRs and the LPS-activation cluster. Trends Immunol. (2002) 23:301–4. doi: 10.1016/S1471-4906(02)02233-0, PMID: 12072369

[35] Dan X Babbar M Moore A Wechter N Tian J Mohanty JG . DNA damage invokes mitophagy through a pathway involving Spata18. Nucleic Acids Res. (2020) 48:6611–23. doi: 10.1093/nar/gkaa393, PMID: 32453416 PMC7337932

[36] Pickrell AM Youle RJ. The roles of PINK1, Parkin, and mitochondrial Fidelity in Parkinson’s disease. Neuron. (2015) 85:257–73. doi: 10.1016/J.NEURON.2014.12.007, PMID: 25611507 PMC4764997

[37] Garbincius JF Elrod JW. Mitochondrial calcium exchange in physiology and disease. Physiol Rev. (2022) 102:893–992. doi: 10.1152/physrev.00041.2020, PMID: 34698550 PMC8816638

[38] Bock FJ Tait SWG. Mitochondria as multifaceted regulators of cell death. Nat Rev Mol Cell Biol. (2020) 21:85–100. doi: 10.1038/s41580-019-0173-8, PMID: 31636403

[39] Wilson DF. Oxidative phosphorylation: regulation and role in cellular and tissue metabolism. J Physiol. (2017) 595:7023–38. doi: 10.1113/JP273839, PMID: 29023737 PMC5709332

[40] Nesci S Rubattu S. UCP2, a member of the mitochondrial uncoupling proteins: an overview from physiological to pathological roles. Biomedicine. (2024) 12:1307. doi: 10.3390/biomedicines12061307, PMID: 38927514 PMC11201685

[41] Vander Heiden MG Cantley LC Thompson CB. Understanding the Warburg effect: the metabolic requirements of cell proliferation. Science. (2009) 324:1029–33. doi: 10.1126/science.1160809, PMID: 19460998 PMC2849637

[42] Weber BZC Arabaci DH Kir S. Metabolic reprogramming in adipose tissue during Cancer Cachexia. Front Oncol. (2022) 12:848394. doi: 10.3389/fonc.2022.848394, PMID: 35646636 PMC9135324

[43] Wang S Zheng Y Yang F Zhu L Zhu X-Q Wang Z-F . The molecular biology of pancreatic adenocarcinoma: translational challenges and clinical perspectives. Signal Transduct Target Ther. (2021) 6:249–23. doi: 10.1038/s41392-021-00659-4, PMID: 34219130 PMC8255319

[44] Zhao J Xie F Yang Y Wang S. Reprogramming of fatty acid metabolism in breast cancer: a narrative review. Transl Breast Cancer Res. (2021) 2. doi: 10.21037/tbcr-20-53

[45] Kuhla B Nürnberg G Albrecht D Görs S Hammon HM Metges CC. Involvement of skeletal muscle protein, glycogen, and fat metabolism in the adaptation on early lactation of dairy cows. J Proteome Res. (2011) 10:4252–62. doi: 10.1021/pr200425h, PMID: 21774562

[46] Wang B Pei J Xu S Liu J Yu J. A glutamine tug-of-war between cancer and immune cells: recent advances in unraveling the ongoing battle. J Exp Clin Cancer Res. (2024) 43:74. doi: 10.1186/s13046-024-02994-0, PMID: 38459595 PMC10921613

[47] Trayhurn P. Hypoxia and adipose tissue function and dysfunction in obesity. Physiol Rev. (2013) 93:1–21. doi: 10.1152/physrev.00017.2012, PMID: 23303904

[48] Reynolds CK Huntington GB Tyrrell HF Reynolds PJ. Net portal-drained visceral and hepatic metabolism of glucose, L-lactate, and nitrogenous compounds in lactating Holstein cows. J Dairy Sci. (1988) 71:1803–12. doi: 10.3168/jds.s0022-0302(88)79749-0, PMID: 2900848

[49] Chouchani ET Kajimura S. Metabolic adaptation and maladaptation in adipose tissue. Nat Metab. (2019) 1:189–200. doi: 10.1038/s42255-018-0021-8, PMID: 31903450 PMC6941795

[50] Colegio OR Chu N-Q Szabo AL Chu T Rhebergen AM Jairam V . Functional polarization of tumour-associated macrophages by tumour-derived lactic acid. Nature. (2014) 513:559–63. doi: 10.1038/nature13490, PMID: 25043024 PMC4301845

[51] Oates EH Antoniewicz MR. 13C-metabolic flux analysis of 3T3-L1 adipocytes illuminates its core metabolism under hypoxia. Metab Eng. (2023) 76:158–66. doi: 10.1016/j.ymben.2023.02.002, PMID: 36758664

[52] Lin Y Bai M Wang S Chen L Li Z Li C . Lactate is a key mediator that links obesity to insulin resistance via modulating cytokine production from adipose tissue. Diabetes. (2022) 71:637–52. doi: 10.2337/db21-0535, PMID: 35044451

[53] Baird GD Lomax MA Symonds HW Shaw SR. Net hepatic and splanchnic metabolism of lactate, pyruvate and propionate in dairy cows in vivo in relation to lactation and nutrient supply. Biochem J. (1980) 186:47–57. doi: 10.1042/bj1860047, PMID: 6989361 PMC1161502

[54] Ahmed K Tunaru S Tang C Müller M Gille A Sassmann A . An autocrine lactate loop mediates insulin-dependent inhibition of lipolysis through GPR81. Cell Metab. (2010) 11:311–9. doi: 10.1016/j.cmet.2010.02.012, PMID: 20374963

[55] Zhang G Hailemariam D Dervishi E Goldansaz SA Deng Q Dunn SM . Dairy cows affected by ketosis show alterations in innate immunity and lipid and carbohydrate metabolism during the dry off period and postpartum. Res Vet Sci. (2016) 107:246–56. doi: 10.1016/j.rvsc.2016.06.012, PMID: 27474003

[56] Horst EA Kvidera SK Baumgard LH. Invited review: the influence of immune activation on transition cow health and performance-a critical evaluation of traditional dogmas. J Dairy Sci. (2021) 104:8380–410. doi: 10.3168/jds.2021-20330, PMID: 34053763

[57] Rico JE Barrientos-Blanco MA. Invited review: ketone biology—the shifting paradigm of ketones and ketosis in the dairy cow. J Dairy Sci. (2024) 107:3367–88. doi: 10.3168/jds.2023-23904, PMID: 38246539

