# Supplementary figures and images for: Bovine adipose mitochondrial adaptation and a potential lactate–ketone toggle in early lactation
Source: Front Vet Sci. 2025 Dec 3;12:1676955. doi: 10.3389/fvets.2025.1676955 (PMC12709676; doi:10.3389/fvets.2025.1676955)

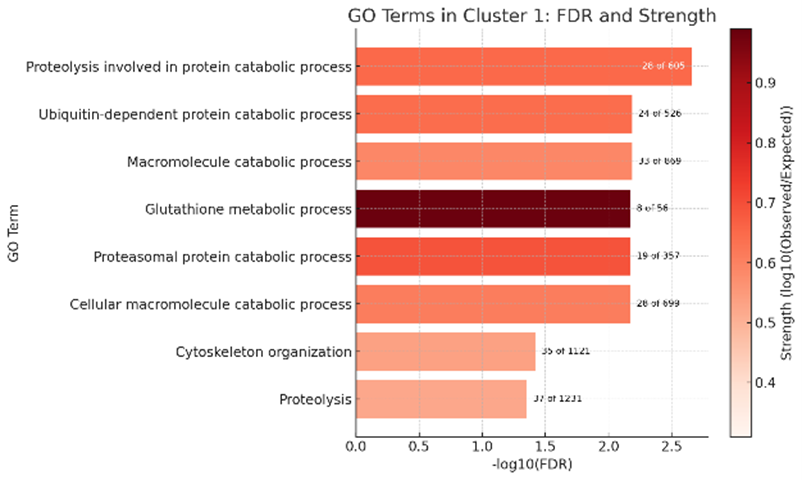

Supplement: SUPPLEMENTARY FIGURE 1 — The top 10 enriched GO terms were ranked by False Discovery Rate (FDR) for Cluster 1 using STRING-db. The bar length represents the-log10(FDR) value, and the colour intensity indicates the enrichment strength (log10(observed/expected)). [file Image_1.tif]

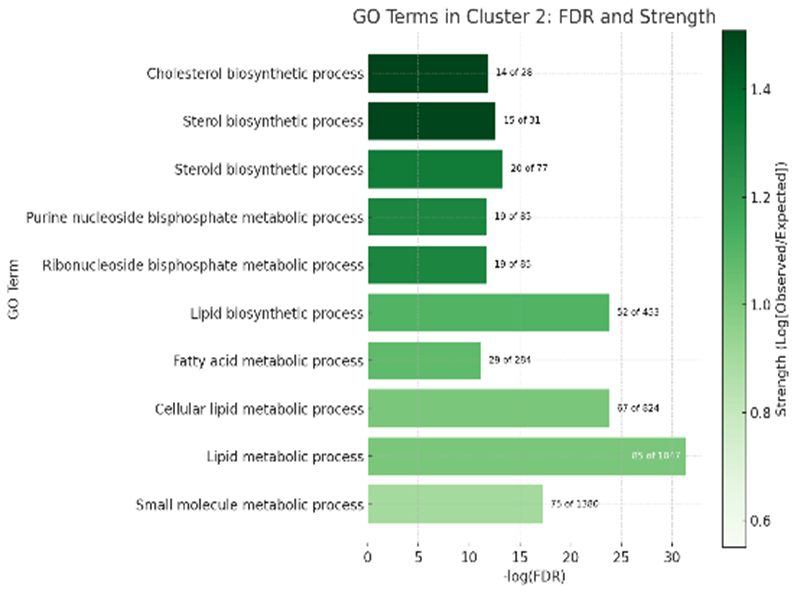

Supplement: SUPPLEMENTARY FIGURE 2 — The top 10 enriched GO terms were ranked by False Discovery Rate (FDR)for Cluster 2 using STRING-db. The bar length represents the-log10(FDR) value, and the colour intensity indicates the enrichment strength (log10(observed/expected)). [file Image_2.tif]

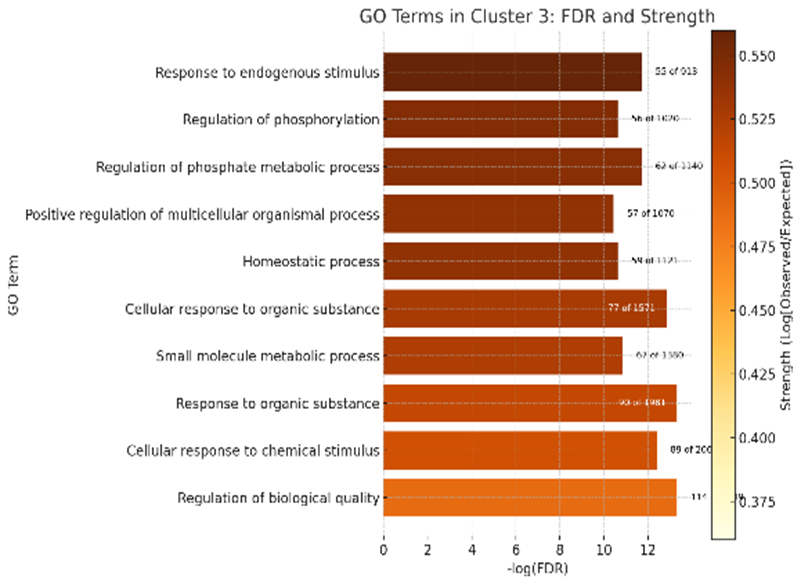

Supplement: SUPPLEMENTARY FIGURE 3 — The top 10 enriched GO terms were ranked by False Discovery Rate (FDR) for Cluster 3 using String-db. The bar length represents the -log10(FDR) value, and the colour intensity indicates the enrichment strength (log10(observed/expected)). [file Image_3.tif]

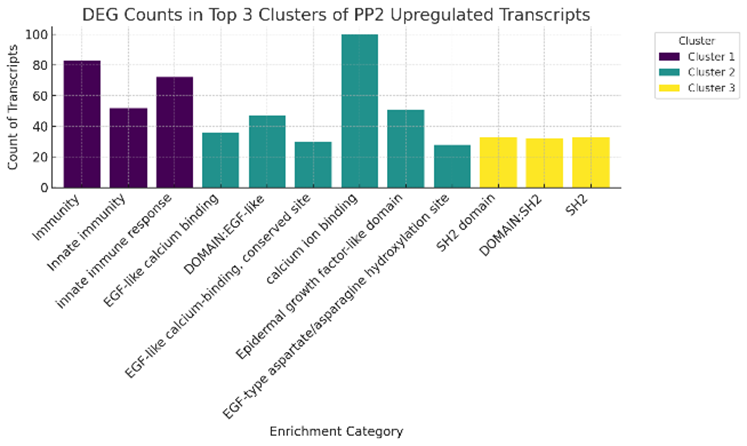

Supplement: SUPPLEMENTARY FIGURE 4 — Functional enrichment of PP2 upregulated transcripts, showing DEG counts across the top three clusters. Bars represent transcript counts per enrichment category, with colours distinguishing clusters. FE Cluster 1 (purple) is enriched in immune-related processes, FE Cluster 2 (teal) in calcium and growth factor binding, and FE Cluster 3 (yellow) in SH2 domain-associated signalling. [file Image_4.tif]

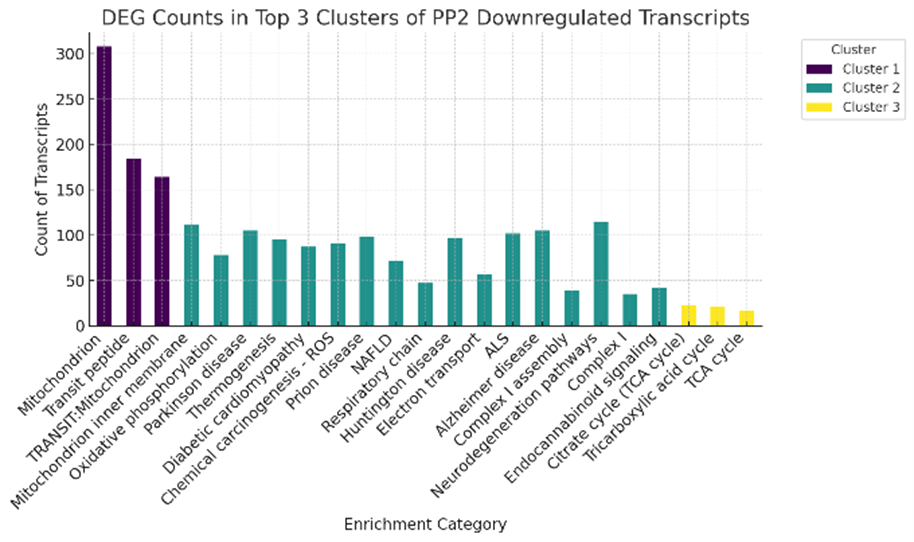

Supplement: SUPPLEMENTARY FIGURE 5 — Functional enrichment of PP2 downregulated transcripts FE Cluster 1 (purple) is enriched in mitochondrial and oxidative metabolism, FE Cluster 2 (teal) in electron transport and neurodegenerative pathways, and FE Cluster 3 (yellow) in TCA cycle-associated processes. [file Image_5.tif]

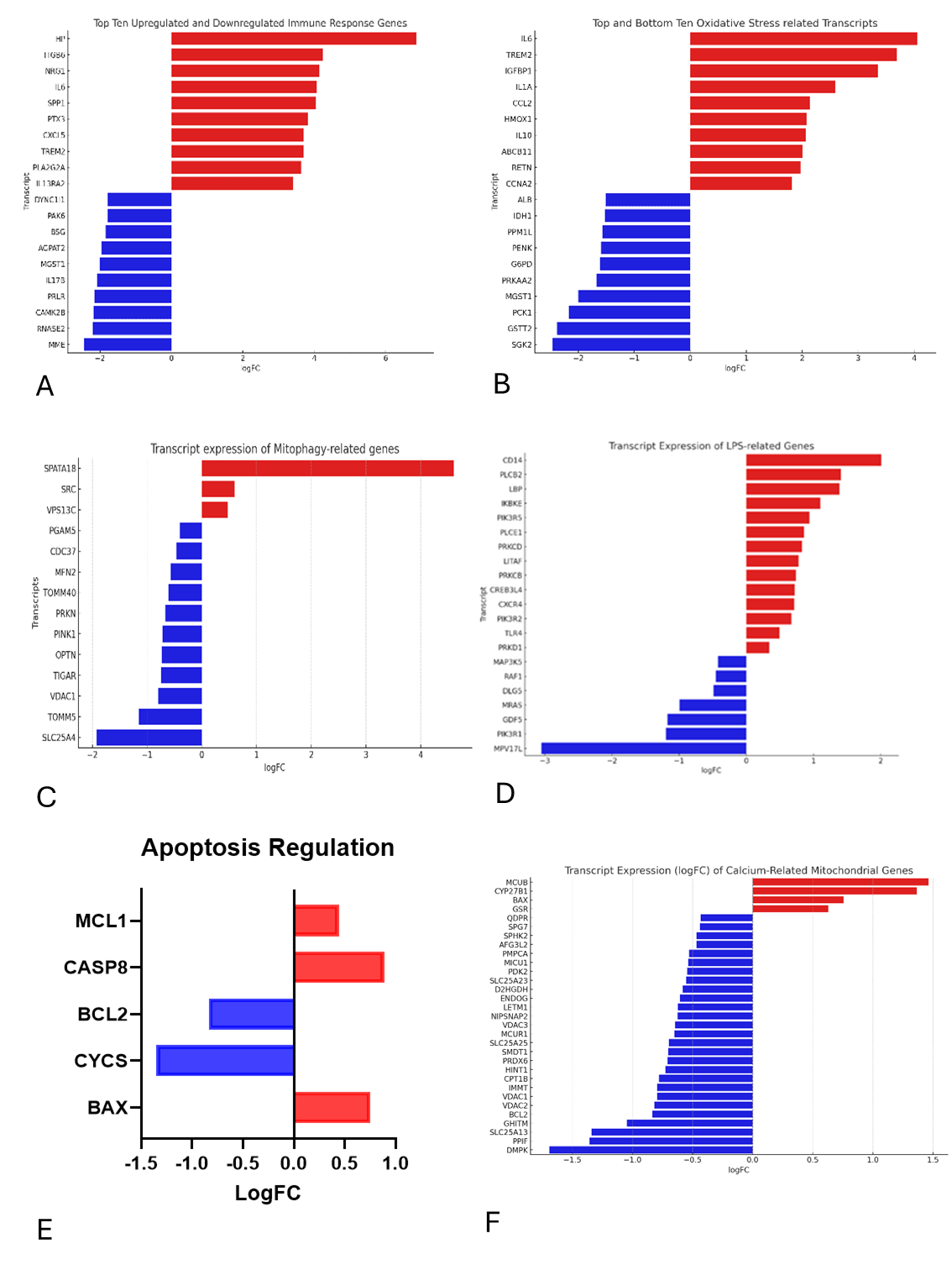

Supplement: SUPPLEMENTARY FIGURE 6 — Upregulated (red) and downregulated (blue) transcripts in PP2 vs. PreP were cross-referenced with pathways from GeneCards. (a) Immune Response. (b) Oxidative Stress. (c) Mitophagy. (d) LPS-related. (e) Apoptosis regulation. (f) Calcium-related mitochondrial genes. [file Image_6.tif]

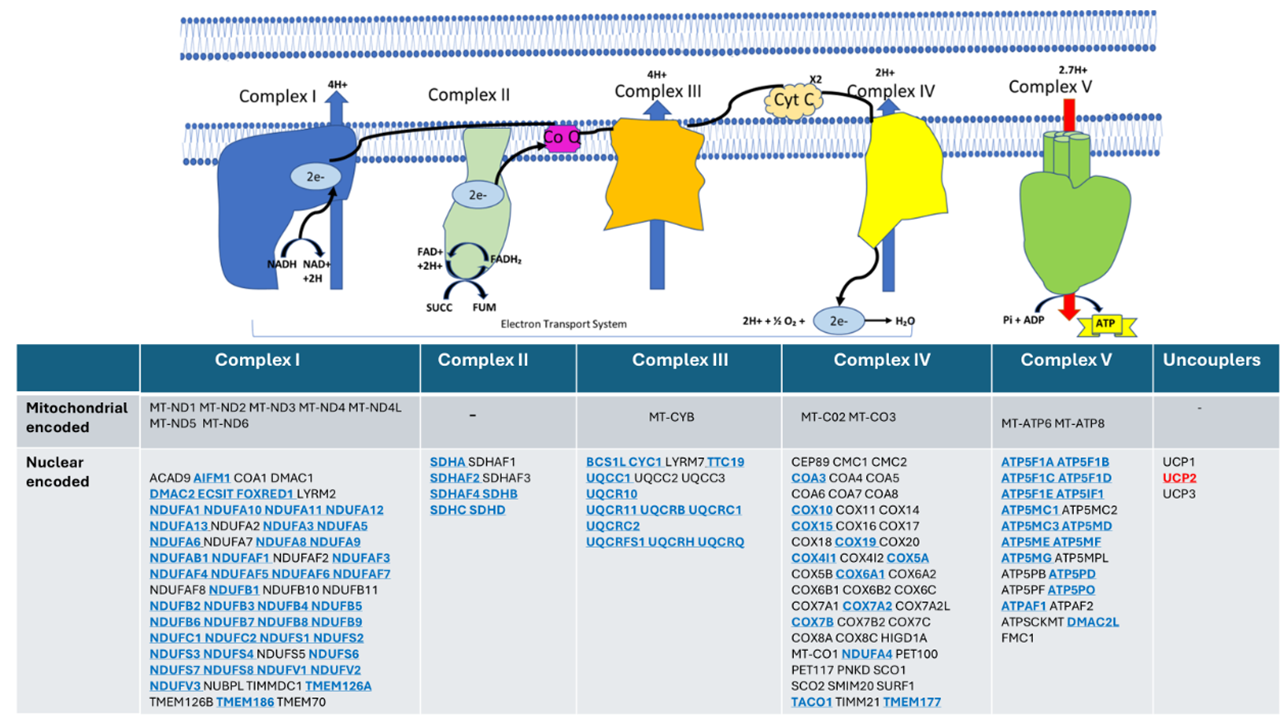

Supplement: SUPPLEMENTARY FIGURE 7 — The electron transport system (complexes I–V) is depicted with associated mitochondrial- and nuclear-encoded genes. Downregulated transcripts (blue) indicate widespread suppression of nuclear-encoded OXPHOS components, and Complex V, while UCP2 (red) highlights an upregulation in uncoupling activity. [file Image_7.tif]

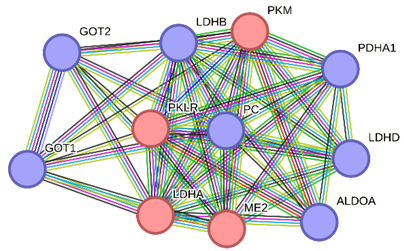

Supplement: SUPPLEMENTARY FIGURE 8 — STRING-db network visualisation depicting protein-protein interactions among key metabolic enzymes linked to pyruvate carboxylase (PC). Red nodes represent upregulated proteins, while blue nodes indicate downregulated proteins. Connectivity between nodes represents functional and regulatory interactions. [file Image_8.tif]

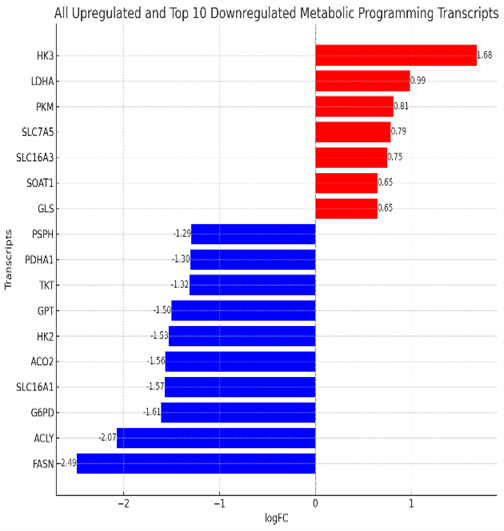

Supplement: SUPPLEMENTARY FIGURE 9 — All upregulated, and the top 10 downregulated transcripts associated with metabolic reprogramming. Red bars indicate upregulated transcripts, while blue bars represent downregulated transcripts, with log fold change (logFC) values shown on the x-axis. [file Image_9.tiff]
