# Supplementary material for: Bovine adipose mitochondrial adaptation and a potential lactate–ketone toggle in early lactation
Source: Front Vet Sci. 2025 Dec 3;12:1676955. doi: 10.3389/fvets.2025.1676955 (PMC12709676; doi:10.3389/fvets.2025.1676955)
Supplement: Supplementary file 13 [file Data_Sheet_1.pdf]

Supplemental Table 1: DEG definition

Immune / Oxidative Stress

DEG	Full Name
HP	Haptoglobin
IL6	Interleukin 6
TREM2	Triggering Receptor Expressed on Myeloid Cells 2
IGFBP3	Insulin Like Growth Factor Binding Protein 3
HMOX1	Heme Oxygenase 1
GSTT1	Glutathione S-Transferase Theta 1
SGK2	Serum/Glucocorticoid Regulated Kinase 2
ALB	Albumin
G6PD	Glucose-6-Phosphate Dehydrogenase
LBP	Lipopolysaccharide Binding Protein
GDF5	Growth Differentiation Factor 5
CXCR4	C-X-C Motif Chemokine Receptor 4
LITAF	Lipopolysaccharide Induced TNF Factor
RNASE2	Ribonuclease A Family Member 2
CAMK2B	Calcium/Calmodulin Dependent Protein Kinase II Beta
PRLR	Prolactin Receptor

Mitophagy / Mitochondrial Quality Control

DEG	Full Name
SPATA18	Spermatogenesis Associated 18
SRC	SRC Proto-Oncogene, Non-Receptor Tyrosine Kinase
VPS13C	Vacuolar Protein Sorting 13 Homolog C
MFN2	Mitofusin 2
PRKN	Parkin RBR E3 Ubiquitin Protein Ligase
PINK1	PTEN Induced Kinase 1
OPTN	Optineurin
VDAC1	Voltage Dependent Anion Channel 1
VDAC2	Voltage Dependent Anion Channel 2
VDAC3	Voltage Dependent Anion Channel 3
SLC25A4	Solute Carrier Family 25 Member 4 (Adenine Nucleotide Translocase 1)

Calcium Transport

Gene Symbol	Full Gene Name
LETM1	LETM1 Domain Containing 1
MCU	Mitochondrial Calcium Uniporter

MCUB	Mitochondrial Calcium Uniporter Dominant Negative Subunit Beta

Apoptosis

Gene Symbol	Full Gene Name
CASP8	Caspase 8
MCL1	MCL1 Apoptosis Regulator, BCL2 Family Member
BAX	BCL2 Associated X Protein

Metabolic Reprogramming

Gene Symbol	Full Gene Name
PC	Pyruvate Carboxylase
PKLR	Pyruvate Kinase L/R
LDHA	Lactate Dehydrogenase A
ME2	Malic Enzyme 2
PKM	Pyruvate Kinase M1/2
PDHA1	Pyruvate Dehydrogenase E1 Subunit Alpha 1
GOT1	Glutamic-Oxaloacetic Transaminase 1
GOT2	Glutamic-Oxaloacetic Transaminase 2
ALDOA	Aldolase, Fructose-Bisphosphate A
HK3	Hexokinase 3
ACO2	Aconitase 2, Mitochondrial
SLC16A1	Solute Carrier Family 16 Member 1 (Monocarboxylate Transporter 1)
HK2	Hexokinase 2
TKT	Transketolase
ACLY	ATP Citrate Lyase
FASN	Fatty Acid Synthase
SLC16A3	Solute Carrier Family 16 Member 3 (Monocarboxylate Transporter 4)
IDH3G	Isocitrate Dehydrogenase 3 (NAD+) Gamma
MDH2	Malate Dehydrogenase 2, Mitochondrial
IDH3B	Isocitrate Dehydrogenase 3 (NAD+) Beta
GLS	Glutaminase
SLC38A1	Solute Carrier Family 38 Member 1
UCP2	Uncoupling Protein 2
SLC38A5	Solute Carrier Family 38 Member 5
