# Supplementary material for: Bovine adipose mitochondrial adaptation and a potential lactate–ketone toggle in early lactation
Source: Front Vet Sci. 2025 Dec 3;12:1676955. doi: 10.3389/fvets.2025.1676955 (PMC12709676; doi:10.3389/fvets.2025.1676955)
Supplement: Supplementary file 14 [file Data_Sheet_2.pdf]

Comparison (Biopsy)	Difference (LS Means)	Std. Error	t Ratio	p-value	95 % CI (Lower, Upper)
PP1 – PP2	0.254	0.093	2.73	0.036	0.015, 0.492
PP1 – PreP	–0.167	0.085	–1.96	0.152	–0.387, 0.051
PP2 – PreP	–0.421	0.092	–4.59	0.0007	–0.656, –0.186
