# Supplementary material for: Bovine adipose mitochondrial adaptation and a potential lactate–ketone toggle in early lactation
Source: Front Vet Sci. 2025 Dec 3;12:1676955. doi: 10.3389/fvets.2025.1676955 (PMC12709676; doi:10.3389/fvets.2025.1676955)
Supplement: Supplementary file 15 [file Data_Sheet_3.pdf]

Parameter	Lactate	Albumin	BHB	Calcium	CHO	Glucose	Magnesium	NEFA	TP	Triglycerides
Lactate	<0.0001	0.7474	0.8257	0.9059	0.5591	0.8982	0.3243	**0.0108**	0.3626	**0.0131**
Albumin	0.7474	<0.0001	0.1795	0.9501	0.5635	0.0827	0.1134	0.6128	0.0840	0.3442
BHB	0.8257	0.1795	<0.0001	0.5634	0.7213	0.0721	0.0941	0.2509	0.7298	0.6765
Calcium	0.9059	0.9501	0.5634	<0.0001	0.9371	0.2626	**0.0168**	0.8276	0.5679	0.0938
CHO	0.5591	0.5635	0.7213	0.9371	<0.0001	0.4238	0.1345	0.2897	0.1529	0.2414
Glucose	0.8982	0.0827	0.0721	0.2626	0.1238	<0.0001	0.4716	0.2534	0.2559	0.8832
Magnesium	0.3243	0.1134	0.0941	**0.0168**	0.1345	0.4716	<0.0001	0.5445	**0.0005**	0.1335
NEFA	**0.0108**	0.6128	0.2509	0.8276	0.2897	0.2534	0.5445	<0.0001	0.2089	0.0962
TP	0.3626	0.0840	0.2769	0.5679	0.1529	0.2559	**0.0005**	0.2089	<0.0001	0.6634
Triglycerides	**0.0131**	0.3442	0.6765	0.0938	0.2414	0.8832	0.1335	0.0962	0.6634	<0.0001
