# Supplementary material for: Bovine adipose mitochondrial adaptation and a potential lactate–ketone toggle in early lactation
Source: Front Vet Sci. 2025 Dec 3;12:1676955. doi: 10.3389/fvets.2025.1676955 (PMC12709676; doi:10.3389/fvets.2025.1676955)
Supplement: Supplementary file 16 [file Data_Sheet_4.pdf]

Parameter	Lactate	Albumin	BHB	Calcium	CHO	Glucose	Magnesium	NEFA	TP	Triglycerides
Lactate	<0.0001	0.1774	0.5684	0.8768	0.0542	0.0979	0.3329	0.0971	**0.0383**	0.7569
Albumin	0.1774	<0.0001	0.3674	0.7547	0.3328	0.4125	0.1375	0.5816	**0.0382**	0.9973
BHB	0.5684	0.3674	<0.0001	0.8847	0.7458	<0.0001	0.9848	0.9355	0.1855	0.4242
Calcium	0.8768	0.7547	0.8847	<0.0001	0.8174	0.8868	**0.0021**	0.2737	0.3871	0.3672
CHO	0.0542	0.3328	0.7458	0.8174	<0.0001	0.2136	0.4126	0.5781	0.8759	0.6151
Glucose	0.0979	0.4125	<0.0001	0.8868	0.2136	<0.0001	0.5884	0.1039	0.1336	0.4231
Magnesium	0.3329	0.1375	0.9848	**0.0021**	0.4126	0.5884	<0.0001	0.9606	**0.0335**	0.3724
NEFA	0.0971	0.5816	0.2737	0.2737	0.5781	0.1039	0.9606	<0.0001	0.3373	0.5629
TP	**0.0383**	**0.0382**	0.1855	0.3871	0.8759	0.1336	**0.0335**	0.3373	<0.0001	0.2244
Triglycerides	0.7569	0.9973	<0.0001	0.3672	0.6151	0.4231	0.3724	0.5629	0.2244	<0.0001
